# Stroke prevention in patients from Latin American countries with non‐valvular atrial fibrillation: Insights from the GARFIELD‐AF registry

**DOI:** 10.1002/clc.23176

**Published:** 2019-04-09

**Authors:** Carlos Jerjes‐Sanchez, Ramon Corbalan, Antonio C. P. Barretto, Hector L. Luciardi, Jagan Allu, Laura Illingworth, Karen S. Pieper, Gloria Kayani, for the GARFIELD‐AF Investigators

**Affiliations:** ^1^ Instituto de Cardiologia y Medicina Vascular TEC Salud, Tecnológico de Monterrey Monterrey Mexico; ^2^ Cardiovascular Division Pontificia Universidad Catolica Santiago Chile; ^3^ Hospital das Clínicas da Faculdad de Medicina da USP Sao Paulo Brazil; ^4^ Universidad Nacional de Tucumán San Miguel de Tucuman Argentina; ^5^ Thrombosis Research Institute London UK; ^6^ Duke Clinical Research Institute Durham North Carolina

**Keywords:** antithrombotic treatment, atrial fibrillation, Latin American, outcomes

## Abstract

**Background:**

Atrial fibrillation (AF) is an important preventable cause of stroke. Anticoagulation (AC) therapy can reduce this risk. However, prescribing patterns and outcomes in patients with non‐valvular AF (NVAF) from Latin American countries are poorly described.

**Methods:**

Using data from the Global Anticoagulant Registry in the FIELD‐AF (GARFIELD‐AF), we examined the stroke prevention strategies and the 1‐year outcomes in patients from four Latin American countries: Argentina, Brazil, Chile, and Mexico.

**Results:**

A total of 4162 patients (2010‐2014) were included in this analysis. At the time of AF diagnosis, 39.9% of patients were prescribed vitamin K antagonists (VKA) ± antiplatelet (AP) therapy, 21.8% non‐VKA oral anticoagulant (NOAC) ± AP, 24.1% AP only and 14.1% no antithrombotic treatment. The proportion of moderate‐high risk patients receiving no AC therapy at participating centers was highest in Mexico (46.4%) and lowest in Chile (14.3%). During 1‐year follow‐up, the rates of all‐cause mortality, stroke/SE and major bleeding were: 5.77 (95% CI) (5.06‐6.56), 1.58 (1.23‐2.02), and 0.99 (0.72‐1.36) and per 100 person‐years, respectively, which are higher than the global rates across all countries in GARFIELD‐AF. Unadjusted rates of all‐cause mortality were highest in Argentina, 6.95 (5.43‐8.90), and lowest in Chile, 4.01 (2.92‐5.52).

**Conclusions:**

GARFIELD‐AF results describes the marked variation in the baseline characteristics and patterns of antithrombotic treatments in patients with NVAF in four Latin American countries. Over one‐third of patients with a moderate‐to‐high risk of stroke received no AC therapy, highlighting the need for improved management of patients according to national guideline.

**Clinical Trial Registration—URL:**

http://www.clinicaltrials.gov. Unique identifier: NCT01090362.

## 1 INTRODUCTION

Atrial fibrillation (AF) is the most common cardiac arrhythmia encountered in clinical practice, with non‐valvular AF (NVAF) comprising the majority of cases.^1^ The prevalence of AF is increasing in both developed and developing countries, owing to an aging population, and this increase is predicted to continue over the coming decades.^2^

Patients with AF have a 5‐fold greater risk of stroke and frequently present in elderly patients. Common risk factors for stroke are hypertension, diabetes, heart failure, smoking, and prior stroke or transient ischemic attack (TIA).^3^, ^4^ In addition to an increased risk of stroke, AF patients frequently present with comorbid myocardial infarction, dementia, and chronic kidney disease.^5^, ^6^

The Global Anticoagulant Registry in the FIELD‐AF (GARFIELD‐AF) is an ongoing prospective, international, multicentre registry of adult patients with newly diagnosed NVAF and one or more additional risk factors for stroke.^7^ Since December 2009, more than 50 000 patients have been enrolled in the registry from 35 countries and patient follow‐up is anticipated to end in the third quarter of 2018. Major goals of the registry are to identify best practices as well as deficiencies in stroke prevention strategies for AF patients and to describe how patient care has evolved over time. As in all registries, there might be substantial regional and intraregional differences among baseline characteristics and use of antithrombotic therapies in patients with new NVAF.^8^

In 2010, the Global Burden of Disease Study estimated that age‐adjusted prevalence of AF in Latin America was 737.9 per 100 000 men and 440.3 per 100 000 women, which is higher than the global average estimated to be 596 per 100 000 men and 373 per 100 000 women.^9^ It is likely that the true prevalence of AF, in general, may be even higher due to asymptomatic or unrecognized AF, which is estimated to account for up to 27% undiagnosed AF patients.^10^

In this paper, we analyzed the baseline characteristics, patterns of antithrombotic therapies and 1‐year outcomes in four Latin American countries, Argentina, Brazil, Chile, and Mexico that participated in the GARFIELD‐AF.

## 2 METHODS

### 2.1 Study design and participants

GARFIELD‐AF is a non‐interventional, observational, worldwide study of NVAF, as described in detail previously.^7^ Patients (≥18 years) were diagnosed with AF according to standard local procedures within the previous 6 weeks and had at least 1 additional factor(s) for stroke as judged by the study investigator. Risk factors were not pre‐specified in the protocol nor were they limited to the components of existing risk stratification schemes. The study excluded patients with a transient, reversible cause of NVAF, and patients for whom follow‐up to 2 years was not envisaged or possible.^7^ Consecutive patients were enrolled prospectively into five sequential cohorts (plus one retrospective cohort of 5000 patients). Investigator sites were randomly selected^11^ and were representative of the care settings in each country.

### 2.2 Data collected at baseline

Baseline data from the patients were collected at the time of diagnosis and included the type of AF, patient demographics, medical history, cardiovascular risk factors, care setting speciality and location, antithrombotic therapy regimen in treated patients, and the main reasons for not providing anticoagulant treatment in untreated patients. International normalized ratio values (INRs) were collected during the first year of follow‐up. Stroke risk was assessed according to CHA_2_DS_2_‐VASc (cardiac failure, hypertension, age ≥ 75 [doubled], diabetes, stroke [doubled]‐vascular disease, age 65‐74 and sex category [female]), and bleeding risk according to HAS‐BLED (hypertension, abnormal renal/liver function, stroke, bleeding history or predisposition, elderly [> 65 years], drugs/alcohol concomitantly) score.^12^, ^13^

Case report forms (CRFs) were submitted to the registry‐coordinating center (Dendrite Clinical Systems Ltd, Henley‐on‐Thames, UK), and the corresponding data were analyzed by an independent statistician. All CRFs were examined by the coordinating center to ascertain completeness and accuracy, and data queries were sent to participating sites. The data used in the study were extracted from the database on October 18, 2017.

### 2.3 Definitions

AC includes vitamin K antagonists (VKAs) and non‐vitamin K antagonist oral anticoagulants (NOACs). The term NOAC includes oral direct factor Xa inhibitors (FXas) and oral direct thrombin inhibitors (DTIs). Vascular disease is defined as peripheral artery disease and/or coronary artery disease with a history of acute coronary syndrome (ACS).^14^ Moderate‐to‐severe chronic kidney disease (CKD) includes stage III to stage V according to the National Kidney Foundation's Kidney Disease Outcomes Quality Initiative guidelines.^15^

### 2.4 Ethics statement

Independent ethics committee and hospital‐based institutional review board approvals were obtained, as necessary, for the registry protocol. The registry is being performed in accordance with the principles of the Declaration of Helsinki, local regulatory requirements, and the International Conference on Harmonization‐Good Pharmacoepidemiological and Clinical Practice guidelines. All patients gave written informed consent to participate. Confidentiality and anonymity of all patients recruited into this registry are maintained at all times.

### 2.5 Statistical analysis

In this analysis, descriptive summaries of patient baseline characteristics were performed for each country, and for all countries taken together. Continuous variables are presented with mean and SDs, and numbers of non‐missing observations are included in the tables and figures. Categorical variables are presented using frequencies and percentages. Baseline differences between countries were evaluated using χ^2^ tests for categorical variables, and Students *t*‐test for continuous variables. Percentages are rounded to one decimal place.

Occurrences of rate mortality are described using the person‐time event rate (per 100 person‐years) and 95% CI. We estimated person‐year rates using a Poisson model, with the number of events as the dependent variable and the log of time as an offset, that is, a covariate with a known coefficient of 1. A log‐rank test was used to evaluate whether at least one country varied in mortality rates compared to the other countries. Stroke/SE and major bleed events were too few for comparison between countries. Due to the large sample size, small differences in results can be statistically significant, thus, clinically important differences are also considered. Data analysis was performed with SAS statistical software, release 9.4 (SAS Institute Inc., Cary, North Carolina).

### 2.6 INRs and time in therapeutic range

Patients receiving VKA therapy at enrolment with ≥3 INR readings and for whom time in therapeutic range (TTR) could be calculated were included in the analysis.^16^ Patient‐level TTR was calculated using linear interpolation between consecutive INR readings according to Rosendaal et al^17^ and using 2.0 to 3.0 as the target INR range.

## 3 RESULTS

### 3.1 Patient population

A total of 4162 patients with NVAF aged ≥18 years were enrolled in GARFIELD‐AF from 113 randomly selected sites in Argentina, Brazil, Chile, and Mexico between August 2010 and July 2016. Nine hundred and fifty‐four patients were enrolled at 24 sites in Argentina, 1065 at 41 sites in Brazil, 987 at 16 sites in Chile and 1156 at 32 sites in Mexico. In Argentina, the majority of AF patients were managed in the private sector (87.1%) compared with a minority of patients from Chile (14.6%). Most patients in GARFIELD‐AF were enrolled by cardiologists (Argentina [88.2%], Brazil [82.3%], Mexico [80.7%]), except in Chile, where the diagnosis and management of AF was shared by cardiologists (47.8%), internal medicine specialists (26.3%) and general practitioners (23.9%).

### 3.2 Risk factors

Baseline characteristics of patients are shown in Table 1. The mean age of patients at the time of diagnosis of AF overall was 69.8 ± 12.0 years, and 52.5% were men. The proportion of patients <65 years of age at the time of diagnosis of AF was highest in Brazil (36.4%), and the proportion of patients ≥75 years of age was highest in Chile (45.1%).

**Table 1:** Baseline characteristics of patients with atrial fibrillation from Argentina, Brazil, Chile, and Mexico and all patients

	Argentina N = 954	Brazil N = 1065	Chile N = 987	Mexico N = 1156	All N = 4162	*P*‐value
Age, mean (SD), years	69.7 (11.2)	67.8 (12.7)	71.5 (11.2)	70.3 (12.3)	69.8 (12.0)	<0.001
Age, n (%)						<0.001
<65	285 (29.9)	388 (36.4)	226 (22.9)	333 (28.8)	1232 (29.6)	
65‐74	332 (34.8)	322 (30.2)	316 (32.0)	345 (29.8)	1034 (31.5)	
≥75	337 (35.3)	355 (33.3)	445 (45.1)	478 (41.3)	1268 (38.6)	
Gender, n (%)						0.002
Male	534 (56.0)	587 (55.1)	504 (51.1)	562 (48.6)	2187 (52.5)	
Female	420 (44.0)	478 (44.9)	483 (48.9)	594 (51.4)	1975 (47.5)	
Ethnicity, n (%)^a^						
Caucasian	285 (30.1)	509 (52.9)	60 (6.1)	15 (1.3)	869 (21.4)	
Hispanic/Latino	663 (69.9)	318 (33.1)	921 (93.3)	1100 (95.2)	3002 (74.1)	
Asian (not Chinese)	0 (0.0)	8 0.8)	1 (0.1)	0 (0.0)	9 (0.2)	
Chinese	0 (0.0)	1 (0.1)	0 (0.0)	0 (0.0)	1 (0.0)	
Afro‐Caribbean	0 (0.0)	33 (3.4)	0 (0.0)	1 (0.1)	34 (0.8)	
Mixed/other	0 (0.0)	93 (9.7)	5 (0.5)	40 (3.5)	138 (3.4)	
Type of AF diagnosed, n (%)						<0.001
New	491 (51.5)	555 (52.1)	370 (37.5)	375 (32.4)	1791 (43.0)	
Paroxysmal	215 (22.5)	271 (25.4)	317 (32.1)	280 (24.2)	1083 (26.0)	
Persistent	69 (7.2)	152 (14.3)	200 (20.3)	202 (17.5)	623 (15.0)	
Permanent	179 (18.8)	87 (8.2)	100 (10.1)	299 (25.9)	665 (16.0)	
Diabetes, n (%)	174 (18.2)	269 (25.3)	253 (25.6)	327 (28.3)	1023 (24.6)	<0.001
Hypercholesterolemia, n (%) b	350 (38.8)	418 (41.8)	361 (40.1)	383 (34.1)	1512 (38.5)	0.002
Current/previous smoker, n (%) c	334 (35.9)	323 (32.2)	281 (30.0)	380 (33.2)	1318 (32.8)	0.021
BMI >30 kg/m^2^, n (%)^d^	289 (39.8)	224 (29.1)	243 (39.5)	331 (31.4)	1087 (34.4)	<0.001
Pulse (bpm), mean(SD)^e^	96.7 (31.1)	89.9 (27.8)	89.2 (27.0)	81.2 (20.7)	88.8 (27.2)	
Systolic blood pressure (mm Hg), mean (SD)^f^	130.5 (18.4)	130.0 (21.5)	135.0 (21.8)	129.9 (19.7)	131.2 (20.5)	
Diastolic blood pressure (mm Hg), mean (SD)^f^	78.2 (12.1)	78.8 (13.0)	79.6 (14.5)	77.3 (11.4)	78.4 (12.7)	
CV medical history, n (%)						
Hypertension, n (%)^g^	770 (81.1)	856 (80.7)	841 (85.5)	892 (77.2)	3359 (81.0)	<0.001
Congestive heart failure	168 (17.6)	274 (25.7)	163 (16.5)	241 (20.8)	846 (20.3)	<0.001
Vascular disease h	146 (15.3)	181 (17.1)	92 (9.3)	181 (15.7)	600 (14.4)	<0.001
Carotid occlusive disease i	20 (2.1)	43 (4.2)	12 (1.2)	31 (2.7)	106 (2.6)	<0.001
CV comorbidities						
Stroke/TIA	71 (7.4)	123 (11.5)	97 (9.8)	191 (16.5)	482 (11.6)	<0.001
Moderate‐to‐severe CKD j	43 (4.5)	96 (9.0)	62 (6.3)	77 (6.7)	278 (6.7)	<0.001
History of bleeding^k^	42 (4.4)	48 (4.5)	26 (2.6)	53 (4.6)	169 (4.1)	0.078
Pulmonary embolism/DVT^l^	17 (1.8)	31 (3.0)	18 (1.8)	35 (3.0)	101 (2.4)	0.099
Systemic embolism^m^	5 (0.5)	13 (1.3)	17 (1.7)	14 (1.2)	49 (1.2)	0.109
Moderate‐heavy alcohol consumption, n (%)^n^	45 (5.3)	96 (9.0)	62 (6.3)	77 (6.7)	278 (6.7)	<0.001
Cirrhosis, n (%)^o^	0 (0.0)	3 (0.3)	4 (0.4)	8 (0.7)	15 (0.4)	0.073
CHA2DS2‐VASc score categories, n(%)						<0.001
0	20 (2.1)	38 (3.7)	30 (3.1)	28 (2.4)	116 (2.8)	
1	115 (12.3)	128 (12.5)	96 (9.9)	110 (9.6)	449 (11.0)	
2	225 (24.0)	177 (17.4)	153 (15.7)	184 (16.0)	739 (18.1)	
3	226 (24.1)	241 (23.6)	233 (23.9)	249 (21.6)	949 (23.3)	
4	203 (21.7)	226 (22.2)	259 (26.6)	310 (26.9)	998 (24.5)	
5	99 (10.6)	111 (10.9)	124 (12.7)	148 (12.9)	482 (11.8)	
6‐9	48 (5.1)	99 (9.7)	78 (8.0)	122 (10.6)	347 (8.5)	

Abbreviations: BMI, body mass index; CKD, chronic kidney disease; CV, cardiovascular; DVT, deep vein thrombosis; TIA. transient ischaemic attack.

a Data unavailable for 109 patients.

b Data unavailable for 237 patients.

c Data unavailable for 148 patients.

d Data unavailable for 1003 patients.

e Data unavailable for 236 patients.

f Data unavailable for 230 patients.

g Data unavailable for 13 patients.

h Data unavailable for 7 patients.

i Data unavailable for 62 patients.

j Data unavailable for 1 patient.

k Data unavailable for 9 patients.

l Data unavailable for 32 patients.

m Data unavailable for 318 patients.

n Data unknown for 99 patients.

o Data unavailable for 82 patients.

One‐quarter of the patients had diabetes mellitus (24.6%), and one‐third were current or previous smokers (32.8%). Obesity (body mass index [BMI] >30 kg/m^2^) was more common in Argentina (39.8%) and Chile (39.5%) than Mexico (31.4%) or Brazil (29.1%).

Across all four countries, common associated risk factors for stroke included hypertension in 81.0% of patients, congestive heart failure (CHF) in 20.3% of patients, and vascular disease in 14.4% of patients. The prevalence of vascular disease at the time of diagnosis of AF was higher in Brazil (17.1%) than Chile (9.3%). More patients from Mexico had experienced a prior stroke or TIA (16.5%) compared with patients from Brazil (11.5%), Chile (9.8%), and Argentina (7.4%). The prevalence of moderate‐to‐severe CKD ranged from 4.5% in Argentina to 9.0% in Brazil (Table 1). Despite these differences, the mean (SD) CHA_2_DS_2_‐VASc score was similar in patients from Argentina, Brazil, Chile, and Mexico: 3.5 (1.6), 3.3 (1.5), 3.2 (1.7), and 3.1 (1.5), respectively.

### 3.3 Antithrombotic treatment for stroke prevention

Upon diagnosis of AF, 39.9% of patients from all four countries were prescribed VKAs ± AP therapy, 21.8% NOACs ± AP therapy and 24.1% AP alone. 14.1% of patients received no antithrombotic treatment (Table 2).

**Table 2:** Anticoagulation treatment patterns at baseline

	Argentina (n = 949)	Brazil (n = 1041)	Chile (n = 976)	Mexico (n = 1140)	All (n = 4106)
VKA, %	26.3	19.8	53.9	18.6	29.1
VKA + AP, %	13.4	9.0	16.1	5.7	10.8
FXaI, %	8.0	16.3	6.9	17.0	12.3
FXaI + AP, %	3.2	4.8	3.3	3.6	3.7
DTI, %	6.0	3.3	1.6	6.9	4.5
DTI + AP, %	2.1	1.4	0.5	1.4	1.3
AP, %^a^	23.2	26.2	10.1	34.9	24.1
No treatment	17.8	19.2	7.6	12.0	14.1

Abbreviations: AP, antiplatelets; DTI, direct thrombin inhibitor; FXaI, Factor Xa inhibitor*;* VKA,vitamin K antagonist.

The *P*‐value for at least one country having a different treatment mix is < 0.001.

a AP alone.

Notable differences in patterns of antithrombotic treatment were found between the countries. The use of NOAC ± AP was higher in Mexico (28.8%) and Brazil (25.8%) than in Argentina (19.3%) and Chile (12.3%). The choice of NOAC prescribed also varied between the countries (Table 2). The proportion of patients who did not receive any antithrombotic treatment was highest in Brazil (19.2%) and Argentina (17.8%) followed by Mexico (12.0%) and Chile (7.6%).

The proportion of patients receiving VKA ± AP was markedly higher in Chile (70.0%) than other countries (Argentina [39.7%], Brazil [28.8%], and Mexico [24.3%]). The proportion of patients receiving APs only was higher in Mexico (34.9%) and lower in Chile (10%).

**Figure 1:**
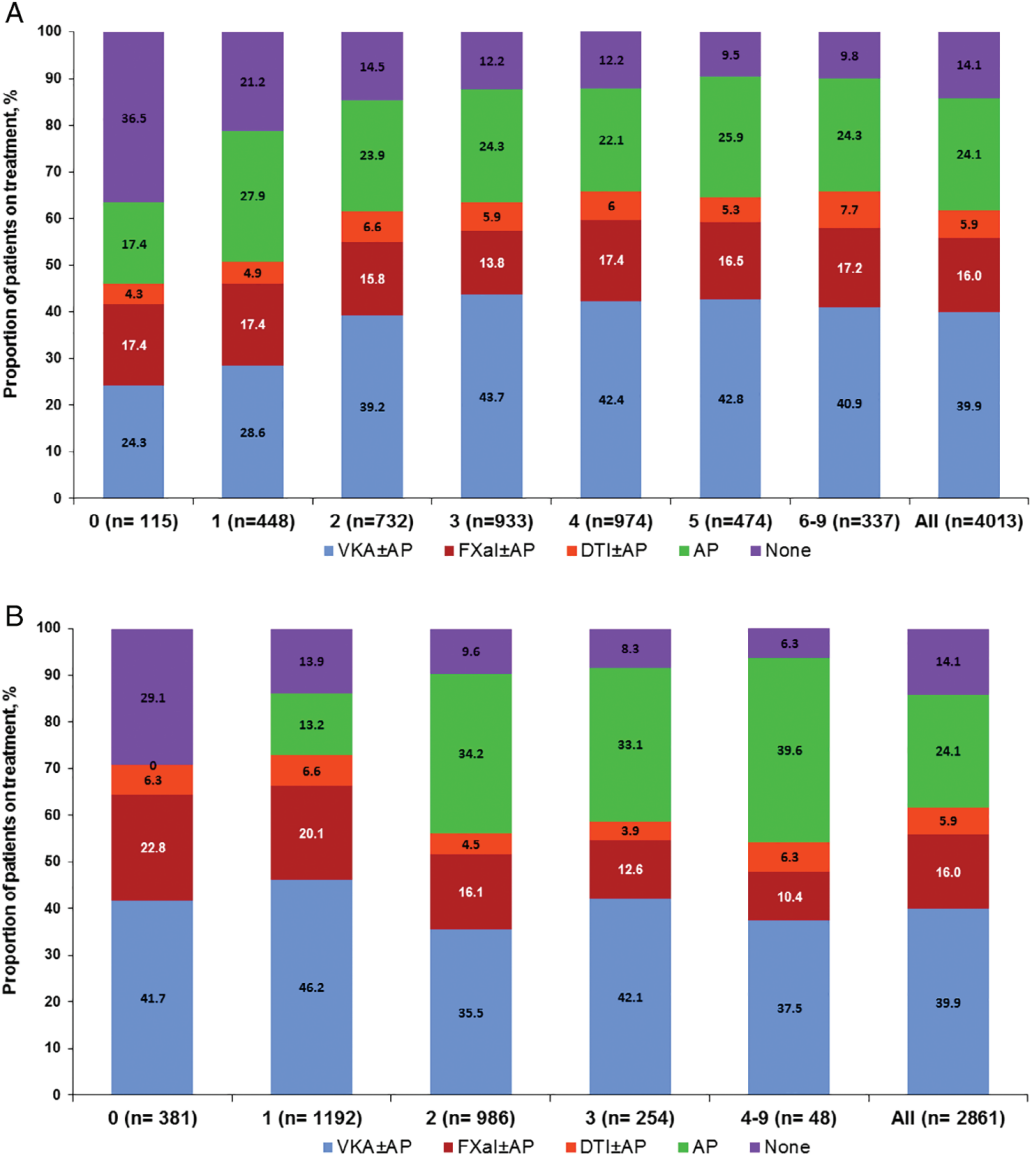
A, Antithrombotic treatment at baseline by CHA_2_DS_2_‐VASc score for all patients in Argentina, Brazil, Chile and Mexico. B, Antithrombotic treatment at baseline by HAS‐BLED score for all patients in Argentina, Brazil, Chile and Mexico. AP, antiplatelet; DTIs, direct thrombin inhibitors; FXas, factor Xa inhibitors; VKAs, vitamin K antagonists

Figure 1A presents the antithrombotic treatment received at baseline by the CHA_2_DS_2_‐VASc score. Overall, 49.9% of patients with a moderate‐to‐severe stroke risk (CHA_2_DS_2_‐VASc score of ≥2) received AC ± AP, 23.8% received AP alone and 12.1% did not receive any antithrombotic treatment. The number of patients who were not prescribed AC ± AP and had a moderate‐high risk of stroke (CHA_2_DS_2_‐VASc score of ≥2) was highest in Mexico (46.4%) and lowest in Chile (14.3%). The proportion of patients with a CHA_2_DS_2_‐VASc score of 0 to 1 who did not receive AC ± AP treatment was highest in Argentina (55.1%).

Figure 1B presents antithrombotic treatment received at baseline by HAS‐BLED score. As the HAS‐BLED score increased, the percentage of patients receiving antithrombotic therapy decreased. Patients with a HAS‐BLED score of ≥4 more frequently received AP (39.6%) compared to patients with a HAS‐BLED score of 0 (0%), 1 (13.2%), 2 (34.2%) and 3(33.1), respectively.

### 3.4 Reasons for anticoagulant therapy was not used

The main reason for not giving an AC to patients at moderate‐to‐high risk of stroke varied between countries but was most frequently the physician's choice (53.8%) (Table S1, Supporting Information).

### 3.5 INR and TTR values

The distribution of INR and TTR values for patients receiving VKA ± AP at baseline is presented in Table S2. For four countries in Latin America, a total of 7636 INR readings were included, with a mean value of 2.3. Overall, 37.2% of the INR values were in the therapeutic range (2.0 to 3.0). A greater proportion of INR values were <2.0 (46.6%) than >3.0 (16.2%). The mean TTR range was 43.4% (SD 25.7%) based on data from 744 patients.

### 3.6 Event rates at 1‐year follow‐up

Overall, event rates per 100 person‐years were highest for all‐cause mortality (5.77; 95% CI: 5.06‐6.56), followed by stroke/SE (1.58; 95% CI: 1.23‐2.02), and major bleeding (0.99; 95% CI: 0.72‐1.36), respectively. Death (n = 228) was most frequently cardiovascular related (46.9%), 35.1% were non‐cardiovascular related, and 18.0% were an undetermined cause. Table 3 shows the unadjusted rates of all‐cause mortality and cause of death in each country.

**Table 3:** Mortality at 1‐year follow‐up

		Cause of death, n (% of total deaths)			
	All‐cause mortality rate (95% CI) per 100‐person years*	Cardiovascular	Non‐cardiovascular	Undetermined	Total number of deaths
Mexico (N = 1187)	5.91 (4.63‐7.53)	33 (50.8%)	16 (24.6%)	16 (24.6%)	65
Brazil (N = 1065)	6.19 (4.83‐7.94)	24 (38.7%)	25 (40.3%)	13 (21.0%)	62
Argentina (N = 954)	6.95 (5.43‐8.90)	28 (44.4%)	26 (41.3%)	9 (14.3%)	63
Chile (N = 987)	4.01 (2.92‐5.52)	22 (57.9%)	13 (34.2%)	3 (7.9%)	38
All (N = 4162)	5.77 (5.06‐6.56)	107 (46.9%)	80 (35.1%)	41 (18.0%)	228

* Log rank *P*‐value = 0.03; The *P*‐value tests whether mortality is different in at least one country.

## 4 DISCUSSION

In this paper, we describe the baseline characteristics, antithrombotic treatment patterns, quality of VKA control and event rates for major clinical outcomes in AF patients from four countries in Latin America, who were enrolled in the GARFIELD‐AF registry.

Even though patients were from the same region, the baseline characteristics and comorbidities were remarkably different for patients from each country. Patients from Chile, for example, were typically older than other in this countries analysis, with a higher incidence of obesity, although these patients had a lower incidence of vascular disease. Interestingly, a history of stroke and TIA was more than twice as frequent in Mexico (16.5%) compared with patients from Argentina (7.4%).

At the time of AF diagnosis, there were also major differences in the choice of AC treatment for stroke prophylaxis in Argentina, Brazil, Chile, and Mexico. The prescription of antithrombotic therapy was highest in Chile where most patients received VKA ± AP (70.0%) rather than NOACs ± AP (12.3%). This was in contrast to Mexico, where prescription of NOACs ± AP was more common (28.8%), with a smaller number of patients receiving VKA ± AP (24.3%). These differences might be related to the differences in patients recruited in countries, differences in enrolling sites and public or private health policies at each country.

Although key guidelines in Latin America, including the Brazilian Society of Cardiology 2009 (BSC),^18^ the Brazilian Cardiogeriatrics Society (BCS)^19^ and the Latin‐American Society of Cerebrovascular Diseases^20^ recommend the use of a VKA as the main oral AC in AF patients at high risk of stroke and stress the importance of assessing the embolic and bleeding risks, the prescription of AC therapy was low and there was still a high use of antiplatelets as an alternation to AC for stroke prevention.

It has been demonstrated that the benefit of AC therapy significantly outweighs the risk of bleeding for AF patients with a CHADS_2_ or CHA_2_DS_2_‐VASc score of ≥2.^21^, ^22^, ^23^ In GARFIELD‐AF, over four‐fifths of patients from Latin America were classified as having a moderate‐to‐high risk of stroke (ie, CHA_2_DS_2_‐VASc score ≥ 2). Of these patients, 35.9% did not receive an anticoagulant. It is widely accepted that AC reduces stroke risk in AF patients; VKAs reduce the risk of stroke by 66% and the risk of death by 28% compared to no therapy.^1^ Despite this evidence, AC remains widely underused in this analysis of four Latin American countries. Moreover, of patients with a low risk of stroke (ie, a CHA_2_DS_2_‐VASc score < 2), approximately half (49.9%) received AC therapy, which indicates overuse of AC in these patients. These figures emphasize the requirement for improved adherence to guidelines with regards to the antithrombotic treatment for stroke prevention. This underuse of OAC is similar to the published reports from other registries in the past decade.^24^

In this registry, the primary reason for patients at high risk of stroke not receiving a VKA was the physician's choice. This may be due to concerns regarding patient's compliance with therapy or risks of bleeding, especially in elderly patients. It has been shown previously that when considering VKA prescription, physicians may overestimate the risk of bleeding and underestimate the benefit of stroke prevention. The underutilization of AC therapy in AF patients may also be perpetuated by misperceptions of the safety and efficacy of aspirin in AF.^25^

The efficacy and safety of VKAs are heavily reliant on the intensity of anticoagulation, as measured by the INR. INR readings during 1‐year follow‐up were analyzed for VKA‐treated patients with ≥3 measurements. Among the measured values in this registry, only 37.2% were in the therapeutic range (2.0‐3.0), whereas 46.6% of the INR values were <2.0 and 16.2% were >3. These figures are in marked contrast to in the findings from clinical trials, comparing NOACs to warfarin, where there was a strong emphasis in accomplishing adequate INRs. Thus, in the ROCKET‐AF trial, 55% of INR values in Latin American patients were in the therapeutic range.^26^ Similarly in the ENGAGE‐AF trial, 62% of Latin American patients were in therapeutic range.^27^ In GARFIELD‐AF, there were almost two‐times as many INR readings of <2, indicating an increased risk of ischemic stroke in the population of patients included in this analysis. Although the use of VKAs varied among the four countries the mean TTRs were similarly low, in contrast to what is recommended in international guidelines.^1^, ^28^ Thus, it is likely that sub‐optimal TTR may contribute to poorer outcomes in patients from four countries of Latin America which is in accordance with the previously published data from GARFIELD‐AF registry.^16^ VKA use requires regular monitoring, patient education, access to coagulation clinics, consistence in the VKAs provided through the public health systems. All these conditions may not be feasible to achieve in many parts of Latin America, due to patient access to healthcare or service costs or low education levels which are contributing to poor patient compliance. Additionally, we only have one presentation of warfarin (5 mg) and acenocoumarin (4 mg) tablets, making dosage difficult for patients. Moreover, the use of generics of different qualities is now common practice in public health systems in Latin America. Thus, sub‐optimal TTR obtained has multiple factors.

It has been suggested that in patients with a TTR <65%, the use of NOACs is likely to provide significant clinical benefits.^29^ Indeed, the use of NOACs in AF patients would eliminate the requirement for frequent monitoring, which may limit the use of AC therapy in many regions of Latin America. Challenges in improving the prescribing of this new class of drugs include: patient education^25^ and lack of adequate public health policies related to the cost of NOACs, which may be unaffordable for many regions in Latin America,^30^ although several pharmaco‐economic studies have shown NOACs to be more cost‐effective than VKAs in Latin America.^31^, ^32^ NOAC use is likely to increase in coming years, given their favorable safety and efficacy profile, as guidelines are updated.^33^ The new Brazilian Guideline from 2016^34^ and Mexican Guidelines,^23^ for example, indicates the use of NOACs in a similar manner to the European guidelines. This has also been stressed by different groups of Latin American investigators.^35^, ^36^

All‐cause mortality was the most frequent major clinical outcome, nearly 4‐fold higher than the rate of stroke/SE, and 6‐fold higher than the rate of major bleeding. Mortality rates from the combined experience: Argentina, Brazil, Chile, and Mexico are higher to those reported in the entire GARFIELD‐AF registry.^37^ However, these numbers are consistent with other reports from Latin America, derived from clinical trials, such as ENGAGE‐AF and ROCKET‐AF.^27^, ^38^ In addition to the poor management of antithrombotic therapies, it is possible that the reasons for the high mortality rates of Latin American patients with AF are related to a higher rate of comorbidities or sociocultural problems, such as difficulties in access to healthcare, lower educational levels, etc. Considering the AC treatment underuse, poor VKA control, and high mortality rate which are challenge for our health systems, developing countries should be warned and achieve their evidence. If our results are reproduced, strategies should be established to improve AF patient care. Whatever the reasons, there is room for improvement in the management of AF patients in Argentina, Brazil, Chile, and Mexico.

## 5 LIMITATIONS

This registry is limited to patients with newly diagnosed AF and the study was mainly conducted by the cardiologists. As with all registries, there may be a bias in the selection of patients and medical centers and so the results may not reflect the experience in all centers in these countries.

## 6 CONCLUSIONS

This paper describes the baseline characteristics and patterns of antithrombotic treatment in patients from four Latin American countries, Argentina, Brazil, Chile, and Mexico. Over one‐third of patients with a moderate‐to‐high risk of stroke received no AC therapy, highlighting the need for better adherence to evidence‐based guidelines on stroke prevention in AF.

## CONFLICT OF INTERESTS

Karen S. Pieper consultant for Thrombosis Research Institute, AstraZeneca, and Bayer. All other authors have reported that they have no relationships relevant to the contents of this paper to disclose.

## Supporting information


**TABLE S1**. Main reasons why OAC was not given to patients with a CHA_2_DS_2_‐VASc score of ≥2
**TABLE S2**. Distribution of INR and TTR values for patients receiving VKA ± AP at baseline

**APPENDIX S1**
