# Supplementary material for: Stroke prevention in patients from Latin American countries with non‐valvular atrial fibrillation: Insights from the GARFIELD‐AF registry
Source: Clin Cardiol. 2019 Apr 9;42(5):553–60. doi: 10.1002/clc.23176 (PMC6522993; doi:10.1002/clc.23176)
Supplement: Supplementary file 1 — TABLE S1. Main reasons why OAC was not given to patients with a CHA2DS2‐VASc score of ≥2 TABLE S2. Distribution of INR and TTR values for patients receiving VKA ± AP at baseline [file CLC-42-553-s002.docx]

**Stroke prevention in patients from Latin America with non-valvular atrial fibrillation: Insights from the GARFIELD-AF registry**

**Table S1. Main reasons why OAC was not given to patients with a CHA_2_DS_2_-VASc score of ≥2**

| **Reason, n (%)** | **Argentina (n=88)** | **Brazil (n=88)** | **Chile (n=32)** | **Mexico (n=242)** | **All (n=450)** |
| --- | --- | --- | --- | --- | --- |
| **Main reason for not using AC** |  |  |  |  |  |
| Alcohol abuse | 0 (0.0) | 0 (0.0) | 1 ( 3.1) | 0 (0.0) | 1 ( 0.2) |
| Already taking AP drugs for other medical condition | 6 (6.8) | 4 ( 4.5) | 1 ( 3.1) | 12 ( 5.0) | 23 ( 5.1) |
| Patient refusal | 6 ( 6.8) | 1 ( 1.1) | 3 ( 9.4) | 18 ( 7.4) | 28 ( 6.2) |
| Previous bleeding event | 3 ( 3.4) | 0 (0.0) | 0 (0.0) | 7 ( 2.9) | 10 ( 2.2) |
| Taking medication contraindicated/ cautioned for use with VKA or AC | 0 (0.0) | 0 (0.0) | 0 (0.0) | 5 ( 2.1) | 5 ( 1.1) |
| Other | 8 ( 9.1) | 11 (12.5) | 2 ( 6.3) | 6 ( 2.5) | 27 ( 6.0) |
| Unknown | 21 (23.9) | 30 (34.1) | 6 (18.8) | 57 (23.6) | 114 (25.3) |
| **Physician's choice** | 44 (50.0) | 42 (47.7) | 19 (59.4) | 137 (56.6) | 242 (53.8) |
| Bleeding risk | 2 ( 2.3) | 7 (8.0) | 8 (25.0) | 14 (5.8) | 31 ( 6.9) |
| Concern over patient compliance | 4 ( 4.5) | 20 (22.7) | 2 ( 6.3) | 14 (5.8) | 40 (8.9) |
| Guideline recommendation | 0 (0.0) | 0 (0.0) | 1 ( 3.1) | 16 ( 6.6) | 17 ( 3.8) |
| Fall risk | 6 ( 6.8) | 3 ( 3.4) | 0 (0.0) | 5 ( 2.1) | 14 ( 3.1) |
| Low risk of stroke | 18 (20.5) | 1 ( 1.1) | 0 (0.0) | 6 ( 2.5) | 25 (5.6) |
| Other | 14 (15.9) | 11 (12.5) | 8 (25.0) | 82 (33.9) | 115 (25.6) |
| Abbreviations: AC=anticoagulant; AP=antiplatelet; CRF=case report form; VKA=vitamin K antagonist. Note: Due to amendments of the CRF, this table only shows data for patients enrolled between December 2009 and June 2013. | | | | | |

**Table S2. Distribution of INR and TTR values for patients receiving VKA±AP at baseline**

|  | **Argentina**  **(N=376)** | **Brazil**  **(N=298)** | **Chile**  **(N=683)** | **Mexico**  **(N=277)** | **All (N=1634)** |
| --- | --- | --- | --- | --- | --- |
| INR, number of readings (%)* |  |  |  |  |  |
| <2.0 | 375 (38.5) | 407 (45.4) | 2592 (48.9) | 183 (39.2) | 3557 (46.6) |
| 2.0–3.0 | 419 (43.0) | 309 (34.5) | 1892 (35.7) | 221 (47.3) | 2841 (37.2) |
| >3.0 | 180 (18.5) | 180 (20.1) | 815 (15.4) | 63 (13.5) | 1238 (16.2) |
| Mean (SD) | 2.4 (1.1) | 2.4 (1.3) | 2.3 (1.1) | 2.3 (1.0) | 2.3 (1.1) |
| TTR, n (%) |  |  |  |  |  |
| < 65 | 76 (69.7) | 78 (78.8) | 387 (83.4) | 45 (62.5) | 586 (78.8) |
| ≥ 65 | 33 (30.3) | 21 (21.2) | 77 (16.6) | 27 (37.5) | 158 (21.2) |
| TTR, mean (SD)** | 49.8 (27.5) | 40.6 (28.4) | 41.7 (22.8) | 48.4 (33.9) | 43.4 (25.7) |
| Abbreviations: INR=international normalized ratio; TTR=time in therapeutic range.  Note: INR values are presented as number of total readings and percentage. TTR was based on 774 patients (Argentina – 109, Brazil – 99, Chile – 464, Mexico – 72), and is presented as mean TTR (SD). * INR p-value: <0.001; **TTR p-value:0.005 | | | | | |
